# Is Samoa Prepared for an Outbreak of COVID-19?

**DOI:** 10.1177/1010539520927283

**Published:** 2020-05-14

**Authors:** Lawal Olatunde Olayemi, Ramona Boodoosingh, Filipina Amosa-Lei Sam

**Affiliations:** 1 School of Medicine, Faculty of Health Sciences, National University of Samoa, Apia, Samoa; 2 School of Nursing, Faculty of Health Sciences, National University of Samoa, Apia, Samoa

There was global panic across all continents when on January 30, 2020, the World Health Organization (WHO) declared the outbreak of the 2019 novel coronavirus disease (COVID-19) as a public health emergency of international concern (PHEIC). The discovery of COVID-19 was linked to the Huanan seafood market in Wuhan, China. As of February 27, 2020, there have been 82 294 confirmed cases globally with the first case in New Zealand.^1^ This constitutes an import risk to neighboring Pacific Island countries including Samoa, which as on February 28, 2020, had no confirmed case of COVID-19.

Because Samoa is an international tourism destination and, coupled with her conservative nature, is highly susceptible to an outbreak of COVID-19 if strict public health regulations are not enforced. This is imperative considering the extent of spread and rapidity of virus transmission, especially among vulnerable populations. The rise of COVID-19 brought back the horror of the last measles epidemic in Samoa and some Pacific Island countries. The gradual decline in the measles epidemic trend was met with a sedulous approach toward case diagnoses, detection, and confirmation. It further stresses the importance of having well-equipped state-of-the-art diagnostic health facilities, public health disease control centers, and highly trained infectious disease personnel. This is a necessity for early disease detection and diagnosis to prevent the occurrence of an epidemic of COVID-19 in Samoa.

It suffices to reason that the small Pacific Island countries could defeat an outbreak of COVID-19 by benchmarking and collaborating with both regional and international multigovernment institutions for technical and financial support. The COVID-19 Joint Incident Management Team (IMT), coordinated by the WHO office in the Pacific, has developed and is implementing a 6-month Pacific Action Plan for COVID-19 Preparedness and Response.^2^ The successful implementation of this action plan is determined by many factors in individual countries, and it mostly involves a cohesive teamwork between the local Ministry of Health and the Joint IMT composed of representatives from WHO and regional donor partners such as Australian Department of Foreign Affairs and Trade (DFAT), New Zealand Ministry of Foreign Affairs and Trade (MFAT), Pacific Community (SPC), and the United Nations Children’s Fund (UNICEF).^2^ The establishment and activation of Samoa’s Health Emergency Operations Centre (HEOC) through the Ministry of Health (MOH) is a step forward for Samoa in preparation for COVID-19 to enhance health sector surveillance, coordination, and provision of situational analysis updates.

Similarly, ongoing are campaigns on epidemic preparedness via leveraging on available resources, capacity building; procurement of laboratory packaging and personal protective equipment (PPE); appropriate identification of isolation and quarantine facilities; and developing case management protocols and strengthening communications to raise public awareness and counter rumors and misinformation.^2^ The MOH has also put in place some public health measures against COVID-19 on the ministry’s website (www.health.gov.ws/coronavirus) to ensure the general public is updated about the ongoing pandemic and, most important, to be aware of prevention and management strategies against COVID-19.
